# Complete genomes and plasmid profiles of five *Lactococcus* isolates from Japanese amberjack (*Seriola quinqueradiata*) and greater amberjack (*Seriola dumerili*) cultured in Japan

**DOI:** 10.1128/mra.01457-25

**Published:** 2026-02-13

**Authors:** Salif Maiga, Riku Ogawa, Masayuki Imajoh

**Affiliations:** 1 Department of Bioresource Production Science, The United Graduate School of Agricultural Sciences, Ehime University 12760, Ehime, Japan; 2 Faculty of Agriculture and Marine Science, Graduate School of Integrated Arts and Science, Kochi University 12888, Kochi, Japan; Montana State University, Bozeman, Montana, USA

**Keywords:** *Lactococcus *isolates, Japanese amberjack, greater amberjack, Japan

## Abstract

The complete genome sequences of *Lactococcus garvieae* KN161025 and *Lactococcus formosensis* strains KN19830, KN201130, KN211206, and KU211113 were determined. Strain KN161025 contained no plasmids, whereas KN19830, KN201130, KN211206, and KU211113 carried two plasmids each.

## ANNOUNCEMENT

In 2024, the annual production of Japanese amberjack (*Seriola quinqueradiata*) and greater amberjack (*Seriola dumerili*) in Japan totaled 104,300 and 23,400 metric tons, respectively, accounting for approximately 50% of the total aquaculture output in the country (1). Meanwhile, lactococcosis has caused substantial economic losses in the *Seriola* aquaculture industry, with estimated damage between 2017 and 2022 accounting for 32.4%–48.4% of the total production value (2). Dead Japanese amberjack and greater amberjack exhibiting lactococcosis symptoms were obtained from farms in Kyushu and Shikoku, respectively, to isolate the causative agents. Five *Lactococcus* isolates were recovered from brain and kidney samples using brain heart infusion (BHI; Becton, Dickinson and Company, Franklin Lakes, NJ, USA) agar supplemented with 1.5% NaCl (BHI-salt) after overnight incubation at 25°C. Among the isolates, KN161025 was identified as *L. garvieae*, and the remaining four isolates (KN19830, KN201130, KN211206, and KU211113) were identified as *L. formosensis* using conventional PCR targeting the *glxR–argS* intergenic region (3).

Frozen stocks of the isolates were cultured on BHI-salt agar plates at 25°C overnight. A single colony of each isolate was selected and incubated with shaking in 10 mL of BHI-salt broth at 25°C overnight. Following high-speed centrifugation, genomic DNA was extracted from the bacterial pellets using the Wizard HMW DNA Extraction Kit (Promega, Madison, WI, USA) and subjected to Illumina and Nanopore sequencing. For Illumina sequencing, genomic DNA was processed using the QIAseq FX DNA Library Kit (Qiagen, Hilden, Germany) to generate an average size of 330 bp. Following adapter ligation, the pooled libraries (20 pM) were sequenced on the Illumina MiSeq platform (MiSeq Reagent Kit v3; 150 cycles, Illumina, San Diego, CA, USA) to generate 75-bp paired-end reads. MiSeq raw reads with Phred quality scores exceeding 30 were retained for assembly using Trim Galore v0.6.5 (4). For Nanopore sequencing, genomic DNA was prepared using the 1D Ligation Sequencing Kit (SQK-LSK109, Oxford Nanopore Technologies, Oxford, UK) with Native Barcoding Expansion (EXP-NBD104) without shearing or size selection to avoid fragmentation. After adapter ligation, the pooled libraries were sequenced on a MinION sequencer (Oxford Nanopore Technologies) with an R9.4.1 flow cell using MinKNOW software v22.05.5. Guppy v4.2.3 in accurate mode was used for base calling. MinION raw reads with quality scores lower than 8 were discarded using NanoFilt v2.8.0 (5). Hybrid assembly of MiSeq and MinION reads was performed using Unicycler v0.4.8 (6) with default parameters, yielding a chromosome for each strain and two plasmids for KN19830, KN201130, KN211206, and KU211113, with error correction, circularization, and rotation completed within the pipeline. Gene annotation, taxonomy verification, and genotyping were performed using the DDBJ Fast Annotation and Submission Tool v1.2.0 (7) and the multilocus sequence typing databases for *L. garvieae* (8). Plasmids were compared to the NCBI databases using BLAST.

As summarized in Table 1, KN201130, KN211206, and KU211113 contained a 27,704-bp plasmid exhibiting 100% query coverage and sequence identity with plasmid pkh2101 from *L. formosensis* KH2101, a known carrier of the *erm*(B) gene and a potential source of antibiotic resistance transmission in aquaculture (9).

**TABLE 1:** Characteristics of the complete genome sequences of five *Lactococcus* isolates

	KN161025	KN19830	KN201130	KN211206	KU211113
Collection date	25 October 2016	30 August 2019	30 November 2020	6 December 2021	13 November 2021
Latitude, longitude	33°22′03.95″N, 133°17′46.28″E	33°22′03.95″N, 133°17′46.28″E	33°22′03.95″N, 133°17′46.28″E	33°22′03.95″N, 133°17′46.28″E	32°15′13.34″N, 130°13′40.89″E
Number of MiSeq reads	8,399,291	2,095,929	1,793,293	1,398,653	1,793,733
Number of MinION reads [N50 value (bp)]	136,782 (8,821)	278,132 (7,667)	1,505,258 (3,020)	212,045 (4,646)	886,710 (3,351)
Number of circular contigs	1	3	3	3	3
Total length (bp)	1,964,765	2,006,817	2,025,226	2,025,255	2,025,208
Chromosome size (bp)	1,964,765	1,988,087	1,988,208	1,988,237	
Plasmids (bp)	No	Two (9,416 and 9,314)	Two (27,704 and 9,314)	Two (27,704 and 9,314)	Two (27,704 and 9,281)
GC content (%)	38.8	38.1	38.1	38.1	38.1
Coverage (×)	891	550	1,335	351	1,017
N50 value (bp)	1,964,765	1,988,087	1,988,208	1,988,237	1,988,223
Number of coding sequences	1,962	1,983	2,007	2,008	2,007
Number of rRNAs	16	16	16	16	16
Number of tRNAs	62	59	59	59	59
ST types	17	56	115	115	115
Whole-genome sequence accession no.	AP043994 for the chromosome	AP043982 for the chromosome, AP043983 and AP043984 for the plasmids	AP043985 for the chromosome, AP043986 and AP043987 for the plasmids	AP043988 for the chromosome, AP043989 and AP043990 for the plasmids	AP043991 for the chromosome, AP043992 and AP043993 for the plasmids
DRA accession no. of Illumina MiSeq and MinION data	DRX705207 for MiSeq data and DRX705208 for MinION data	DRX705195 for MiSeq data and DRX705199 for MinION data	DRX705196 for MiSeq data and DRX705200 for MinION data	DRX705197 for MiSeq data and DRX705201 for MinION data	DRX705198 for MiSeq data and DRX705202 for MinION data
Highest average nucleotide identity value	99.98% with *L*. *garvieae* ATCC 49156	98.15% with *L*. *formosensis* NBRC 109475	98.15% with *L*. *formosensis* NBRC 109475	98.15% with *L*. *formosensis* NBRC 109475	98.15% with *L*. *formosensis* NBRC 109475

## Data Availability

The complete genome sequences and the raw sequencing data generated using the Illumina MiSeq and MinION platforms for the five *Lactococcus* isolates are available at the National Center for Biotechnology Information and the DDBJ Sequence Read Archive, respectively, under the accession numbers listed in Table 1.
